# Crystal structure of tris­(1,10-phenanthroline-κ^2^ *N*,*N*′)iron(II) bis­[bis­(tri­fluoro­methyl­sulfon­yl)imide] monohydrate

**DOI:** 10.1107/S2056989014026966

**Published:** 2015-01-01

**Authors:** Kazunori Teramoto, Takeshi Kawasaki, Toshikazu Nishide, Yasuhisa Ikeda

**Affiliations:** a Department of Chemical Biology and Applied Chemistry, College of Engineering, Nihon University, 1 Nakagawara Tokusada Tamura, Koriyama 963-8642, Japan; b Department of Chemistry, Faculty of Science, Toho University, 2-2-1 Miyama, Funabashi, Chiba 274-8510, Japan; c Research Laboratory for Nuclear Reactors, Tokyo Institute of Technology, 2-12-1-N1-34 Ookayama, Meguro-ku, Tokyo 152-8550, Japan

**Keywords:** crystal structure, 1,10-phenanthroline, iron(II) complex, complex salt, bis­(tri­fluoro­methyl­sulfon­yl)imide, low-spin *d*^6^ Fe^II^ ions, hydrogen bonding

## Abstract

The crystal structure of the title complex, [Fe(C_12_H_8_N_2_)_3_][(CF_3_SO_2_)_2_N]_2_·H_2_O, is constructed by one octa­hedral [Fe(phen)_3_]^2+^ (phen = 1,10-phenanthroline) cation (point group symmetry 2), two Tf_2_N^−^ [bis­(tri­fluoromethyl­sulfon­yl)imide] anions, and one water mol­ecule of crystallization (point group 2). The Fe—N bond lengths are indicative of a *d* ^6^ low-spin state for the Fe^II^ ion in the complex. The dihedral angle between the phen ligands in the cation is 87.64 (6)°. The Tf_2_N^−^ counter-anion is non-coordinating, with the –CF_3_ groups arranged in a *trans* fashion with respect to each other, leading to an *anti,anti* conformation of the –CF_3_ groups and –SO_2_N– moieties relative to the S—C bonds. The water mol­ecule of crystallization connects two O atoms of the Tf_2_N^−^ anions through weak hydrogen bonds. C—H⋯O hydrogen-bonding inter­actions are also observed, consolidating the packing of the mol­ecules into a three-dimensional network structure.

## Related literature   

For the synthesis of the anhydrous title complex, see: Teramoto *et al.* (2014 ▸). For typical Fe—N bond lengths of low-spin *d* ^6^ Fe^II^ ions, see: Deng *et al.* (2001 ▸); Setifi *et al.* (2013 ▸). Crystal structures of complexes with the [Fe(phen)_3_]^2+^ cation were reported by Koh (1994 ▸), Potočňák *et al.* (2014 ▸) and Zhong (2012 ▸). In the crystal structure of the ionic liquid choline bis­(tri­fluoro­methyl­sulfon­yl)imide (Nockemann *et al.*, 2009 ▸), the free Tf_2_N^−^ anion has a similar conformation to that in the title compound.
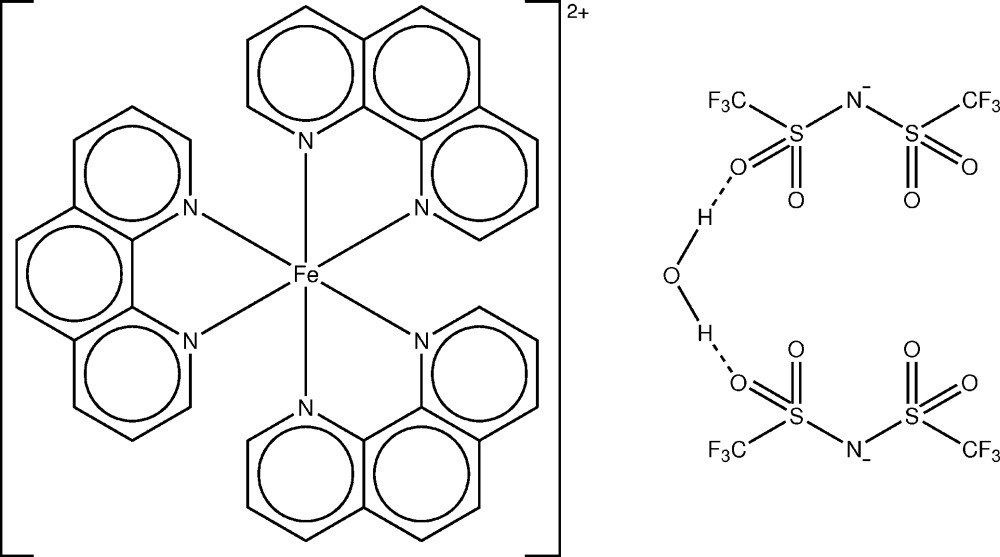


## Experimental   

### Crystal data  

[Fe(C_12_H_8_N_2_)_3_](C_2_F_6_NO_4_S_2_)_2_·H_2_O
*M* *_r_* = 1174.78
Monoclinic, 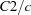

*a* = 20.7745 (15) Å
*b* = 16.0107 (12) Å
*c* = 13.3084 (10) Å
β = 91.657 (1)°
*V* = 4424.7 (6) Å^3^
*Z* = 4
Mo *K*α radiation
μ = 0.65 mm^−1^
*T* = 100 K
0.42 × 0.11 × 0.10 mm

### Data collection  

Bruker APEXII CCD area-detector diffractometer
Absorption correction: multi-scan (*SADABS*; Bruker, 2007 ▸) *T*_min_ = 0.773, *T*_max_ = 0.938
13659 measured reflections
4910 independent reflections
3247 reflections with *I* > 2σ(*I*)
*R*_int_ = 0.065

### Refinement  

*R*[*F* ^2^ > 2σ(*F* ^2^)] = 0.048
*wR*(*F* ^2^) = 0.122
*S* = 1.02
4910 reflections
338 parameters
2 restraints
H atoms treated by a mixture of independent and constrained refinement
Δρ_max_ = 0.71 e Å^−3^
Δρ_min_ = −0.90 e Å^−3^

### 

Data collection: *APEX2* (Bruker, 2007 ▸); cell refinement: *SAINT* (Bruker, 2007 ▸); data reduction: *SAINT*; program(s) used to solve structure: *SHELXS97* (Sheldrick, 2008 ▸); program(s) used to refine structure: *SHELXL97* (Sheldrick, 2008 ▸); molecular graphics: *SHELXTL* (Sheldrick, 2008 ▸); software used to prepare material for publication: *SHELXTL* and *PLATON* (Spek, 2009 ▸).

## Supplementary Material

Crystal structure: contains datablock(s) I, global. DOI: 10.1107/S2056989014026966/wm5100sup1.cif

Structure factors: contains datablock(s) I. DOI: 10.1107/S2056989014026966/wm5100Isup2.hkl

**Fig. 1.**
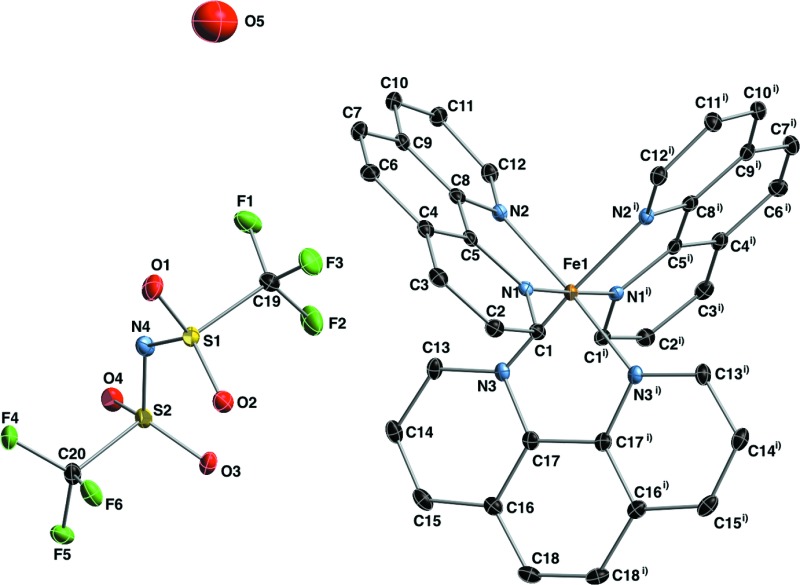
View of the [Fe(phen)3]2+, Tf2N- and H2O molecular units. Displacement ellipsoids are represented at the 30% probability level. Hydrogen atoms were omitted for clarity. [Symmetry code: i) -x + 1,y,-z + 1/2.]

Click here for additional data file.3 2+ 2 − 2 x y z . DOI: 10.1107/S2056989014026966/wm5100fig1.tif
View of the [Fe(phen)_3_]^2+^, Tf_2_N^−^ and H_2_O mol­ecular units. Displacement ellipsoids are represented at the 30% probability level. Hydrogen atoms were omitted for clarity. [Symmetry code: i) −*x* + 1,*y*,-*z* + 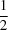.]

**Fig. 2.**
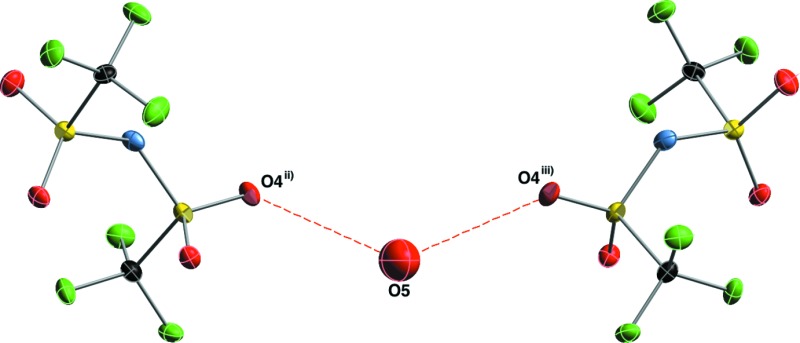
Hydrogen bonds between the H2O molecule and Tf2N- anions. [Symmetry codes: ii) x - 1/2,y - 1/2,z, iii) -x + 3/2, y - 1/2, -z + 3/2.]

Click here for additional data file.2 2 − x y z x y z . DOI: 10.1107/S2056989014026966/wm5100fig2.tif
Hydrogen bonds between the H_2_O mol­ecule and Tf_2_N^−^ anions. [Symmetry codes: ii) *x* − 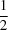,*y* − 1/2,*z*, iii) −*x* + 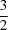, *y* − 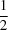, −*z* + 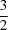.]

CCDC reference: 1038289

Additional supporting information:  crystallographic information; 3D view; checkCIF report

## Figures and Tables

**Table 1:** Selected bond lengths ()

Fe1N1	1.977(3)
Fe1N2	1.974(3)
Fe1N3	1.966(3)

**Table 2:** Hydrogen-bond geometry (, )

*D*H*A*	*D*H	H*A*	*D* *A*	*D*H*A*
O5H5*W*O4^i^	0.88(1)	2.23(6)	2.963(4)	141(7)
C2H2O3^ii^	0.95	2.50	3.433(4)	166
C14H14O2	0.95	2.53	3.481(5)	174

Symmetry codes: (i) 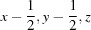; (ii) 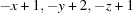.

## supplementary crystallographic information

### S1. Experimental

Red powders of [Fe(phen)_3_](Tf_2_N)_2_ were synthesized as described in the literature by Teramoto *et al.* (2014). The title complex was crystallized by cooling a hot concentrated aqueous solution of [Fe(phen)_3_](Tf_2_N)_2_ .

### S2. Refinement

The H atom of the water molecule was located in a difference map and was refined by applying a restraint for the O—H bond length (0.85 (1) Å). The remaining H atoms were positioned geometrically with C—H = 0.95 Å. All H atoms were constrained to ride on their parent atoms with *U*_iso_(H) = 1.2*U*_eq_(C) or *U*_iso_(H) = 1.5 *U*_eq_(O).

### Crystal data

**Table:**

[Fe(C_12_H_8_N_2_)_3_](C_2_F_6_NO_4_S_2_)_2_·H_2_O	*F*(000) = 2368
*M**_r_* = 1174.78	*D*_x_ = 1.764 Mg m^−^^3^
Monoclinic, *C*2/*c*	Mo *K*α radiation, λ = 0.71073 Å
Hall symbol: -C 2yc	Cell parameters from 1623 reflections
*a* = 20.7745 (15) Å	θ = 2.2–23.5°
*b* = 16.0107 (12) Å	µ = 0.65 mm^−^^1^
*c* = 13.3084 (10) Å	*T* = 100 K
β = 91.657 (1)°	Block, red
*V* = 4424.7 (6) Å^3^	0.42 × 0.11 × 0.10 mm
*Z* = 4	

### Data collection

**Table:**

Bruker APEXII CCD area-detector diffractometer	4910 independent reflections
Radiation source: fine-focus sealed tube	3247 reflections with *I* > 2σ(*I*)
Graphite monochromator	*R*_int_ = 0.065
Detector resolution: 8.333 pixels mm^-1^	θ_max_ = 27.2°, θ_min_ = 1.6°
phi and ω scans	*h* = −26→24
Absorption correction: multi-scan (*SADABS*; Bruker, 2007)	*k* = −17→20
*T*_min_ = 0.773, *T*_max_ = 0.938	*l* = −17→16
13659 measured reflections	

### Refinement

**Table:**

Refinement on *F*^2^	Primary atom site location: structure-invariant direct methods
Least-squares matrix: full	Secondary atom site location: difference Fourier map
*R*[*F*^2^ > 2σ(*F*^2^)] = 0.048	Hydrogen site location: inferred from neighbouring sites
*wR*(*F*^2^) = 0.122	H atoms treated by a mixture of independent and constrained refinement
*S* = 1.02	*w* = 1/[σ^2^(*F*_o_^2^) + (0.0533*P*)^2^ + 0.4369*P*] where *P* = (*F*_o_^2^ + 2*F*_c_^2^)/3
4910 reflections	(Δ/σ)_max_ = 0.001
338 parameters	Δρ_max_ = 0.71 e Å^−^^3^
2 restraints	Δρ_min_ = −0.90 e Å^−^^3^

### Special details

**Table:**

Geometry. All e.s.d.'s (except the e.s.d. in the dihedral angle between two l.s. planes) are estimated using the full covariance matrix. The cell e.s.d.'s are taken into account individually in the estimation of e.s.d.'s in distances, angles and torsion angles; correlations between e.s.d.'s in cell parameters are only used when they are defined by crystal symmetry. An approximate (isotropic) treatment of cell e.s.d.'s is used for estimating e.s.d.'s involving l.s. planes.
Refinement. Refinement of *F*^2^ against ALL reflections. The weighted *R*-factor *wR* and goodness of fit *S* are based on *F*^2^, conventional *R*-factors *R* are based on *F*, with *F* set to zero for negative *F*^2^. The threshold expression of *F*^2^ > σ(*F*^2^) is used only for calculating *R*-factors(gt) *etc*. and is not relevant to the choice of reflections for refinement. *R*-factors based on *F*^2^ are statistically about twice as large as those based on *F*, and *R*- factors based on ALL data will be even larger.

### Fractional atomic coordinates and isotropic or equivalent isotropic displacement parameters (Å^2^)

**Table:**

	*x*	*y*	*z*	*U*_iso_*/*U*_eq_	
Fe1	0.5000	0.81145 (4)	0.2500	0.01247 (17)	
S1	0.70284 (4)	0.87060 (6)	0.73999 (6)	0.0194 (2)	
S2	0.81435 (4)	0.96272 (6)	0.78549 (6)	0.0190 (2)	
N1	0.41901 (12)	0.80991 (17)	0.32441 (19)	0.0128 (6)	
N2	0.52464 (12)	0.72317 (17)	0.34722 (19)	0.0136 (6)	
N3	0.53087 (12)	0.90330 (18)	0.33681 (19)	0.0141 (6)	
N4	0.77157 (14)	0.88188 (19)	0.7924 (2)	0.0231 (7)	
O1	0.66576 (12)	0.81717 (18)	0.80049 (19)	0.0320 (7)	
O2	0.67338 (11)	0.94387 (16)	0.69810 (18)	0.0243 (6)	
O3	0.80627 (11)	1.01191 (16)	0.69622 (17)	0.0235 (6)	
O4	0.87849 (11)	0.94170 (17)	0.81960 (19)	0.0264 (6)	
O5	0.5000	0.5160 (4)	0.7500	0.102 (2)	
H5W	0.4670 (6)	0.4824 (12)	0.742 (7)	0.153*	
F1	0.75397 (11)	0.73840 (14)	0.65849 (17)	0.0369 (6)	
F2	0.75628 (11)	0.84768 (16)	0.56672 (17)	0.0409 (6)	
F3	0.66724 (10)	0.78257 (14)	0.58392 (17)	0.0343 (6)	
F4	0.78834 (11)	0.98981 (14)	0.97358 (15)	0.0318 (6)	
F5	0.82027 (10)	1.09733 (13)	0.89196 (16)	0.0281 (5)	
F6	0.72349 (9)	1.05100 (14)	0.86804 (15)	0.0283 (5)	
C1	0.36704 (15)	0.8593 (2)	0.3148 (2)	0.0150 (7)	
H1	0.3646	0.8974	0.2601	0.018*	
C2	0.31626 (16)	0.8573 (2)	0.3816 (2)	0.0175 (8)	
H2	0.2807	0.8941	0.3725	0.021*	
C3	0.31802 (15)	0.8018 (2)	0.4603 (3)	0.0177 (8)	
H3	0.2841	0.8008	0.5067	0.021*	
C4	0.37042 (16)	0.7465 (2)	0.4717 (2)	0.0160 (7)	
C5	0.42054 (15)	0.7547 (2)	0.4026 (2)	0.0143 (7)	
C6	0.37791 (16)	0.6843 (2)	0.5492 (3)	0.0190 (8)	
H6	0.3445	0.6769	0.5956	0.023*	
C7	0.43081 (16)	0.6365 (2)	0.5575 (2)	0.0197 (8)	
H7	0.4339	0.5955	0.6090	0.024*	
C8	0.47740 (15)	0.7066 (2)	0.4134 (2)	0.0153 (7)	
C9	0.48305 (16)	0.6464 (2)	0.4898 (2)	0.0164 (7)	
C10	0.54083 (16)	0.5999 (2)	0.4945 (3)	0.0206 (8)	
H10	0.5475	0.5585	0.5449	0.025*	
C11	0.58709 (17)	0.6152 (2)	0.4258 (2)	0.0193 (8)	
H11	0.6255	0.5830	0.4268	0.023*	
C12	0.57795 (16)	0.6777 (2)	0.3545 (2)	0.0173 (8)	
H12	0.6114	0.6884	0.3090	0.021*	
C13	0.56412 (15)	0.9013 (2)	0.4249 (2)	0.0177 (8)	
H13	0.5741	0.8486	0.4542	0.021*	
C14	0.58467 (16)	0.9738 (2)	0.4751 (3)	0.0199 (8)	
H14	0.6086	0.9698	0.5368	0.024*	
C15	0.57025 (16)	1.0499 (2)	0.4352 (3)	0.0209 (8)	
H15	0.5840	1.0993	0.4692	0.025*	
C16	0.53488 (16)	1.0558 (2)	0.3435 (3)	0.0178 (8)	
C17	0.51693 (15)	0.9803 (2)	0.2973 (2)	0.0153 (7)	
C18	0.51659 (16)	1.1325 (2)	0.2942 (3)	0.0214 (8)	
H18	0.5282	1.1843	0.3246	0.026*	
C19	0.72140 (18)	0.8058 (2)	0.6315 (3)	0.0236 (8)	
C20	0.78431 (17)	1.0287 (2)	0.8856 (3)	0.0213 (8)	

### Atomic displacement parameters (Å^2^)

**Table:**

	*U*^11^	*U*^22^	*U*^33^	*U*^12^	*U*^13^	*U*^23^
Fe1	0.0125 (3)	0.0142 (4)	0.0107 (3)	0.000	0.0000 (3)	0.000
S1	0.0223 (5)	0.0212 (5)	0.0148 (4)	0.0016 (4)	−0.0001 (4)	0.0002 (4)
S2	0.0201 (5)	0.0208 (5)	0.0161 (4)	0.0048 (4)	−0.0013 (4)	−0.0022 (4)
N1	0.0138 (14)	0.0118 (15)	0.0125 (14)	−0.0030 (12)	−0.0016 (11)	−0.0011 (11)
N2	0.0119 (14)	0.0160 (16)	0.0128 (14)	0.0010 (12)	−0.0006 (11)	−0.0017 (12)
N3	0.0112 (13)	0.0188 (16)	0.0124 (14)	0.0001 (12)	0.0017 (11)	−0.0010 (12)
N4	0.0255 (16)	0.0209 (18)	0.0223 (16)	0.0033 (14)	−0.0104 (14)	−0.0022 (13)
O1	0.0329 (15)	0.0405 (19)	0.0229 (14)	−0.0055 (13)	0.0053 (12)	0.0070 (13)
O2	0.0242 (13)	0.0233 (16)	0.0249 (14)	0.0049 (11)	−0.0071 (11)	−0.0013 (11)
O3	0.0247 (13)	0.0297 (16)	0.0162 (13)	0.0038 (11)	0.0001 (11)	0.0037 (11)
O4	0.0198 (13)	0.0316 (17)	0.0276 (14)	0.0076 (12)	−0.0043 (11)	−0.0029 (12)
O5	0.088 (5)	0.098 (6)	0.120 (6)	0.000	−0.005 (5)	0.000
F1	0.0399 (14)	0.0241 (14)	0.0461 (15)	0.0071 (11)	−0.0105 (12)	−0.0124 (11)
F2	0.0504 (15)	0.0476 (17)	0.0257 (12)	−0.0160 (12)	0.0163 (12)	−0.0074 (11)
F3	0.0352 (13)	0.0325 (14)	0.0343 (13)	−0.0027 (11)	−0.0124 (11)	−0.0101 (11)
F4	0.0465 (14)	0.0333 (14)	0.0153 (11)	0.0019 (11)	−0.0021 (10)	0.0005 (10)
F5	0.0301 (12)	0.0205 (13)	0.0334 (13)	−0.0013 (10)	−0.0050 (10)	−0.0065 (10)
F6	0.0210 (11)	0.0354 (14)	0.0283 (12)	0.0071 (10)	−0.0019 (9)	−0.0127 (10)
C1	0.0140 (16)	0.0155 (19)	0.0152 (17)	0.0005 (14)	−0.0026 (14)	−0.0021 (14)
C2	0.0137 (17)	0.018 (2)	0.0201 (18)	0.0011 (14)	−0.0022 (14)	−0.0013 (15)
C3	0.0131 (16)	0.020 (2)	0.0195 (18)	−0.0058 (14)	0.0033 (14)	−0.0044 (15)
C4	0.0156 (17)	0.017 (2)	0.0156 (17)	−0.0053 (14)	−0.0012 (14)	−0.0030 (14)
C5	0.0174 (17)	0.0144 (19)	0.0111 (16)	−0.0036 (14)	−0.0003 (14)	−0.0037 (13)
C6	0.0212 (18)	0.0158 (19)	0.0201 (18)	−0.0044 (15)	0.0036 (15)	−0.0002 (15)
C7	0.0262 (19)	0.020 (2)	0.0136 (17)	−0.0033 (16)	0.0047 (15)	0.0024 (15)
C8	0.0170 (17)	0.0142 (19)	0.0145 (17)	−0.0051 (14)	−0.0006 (14)	−0.0030 (14)
C9	0.0202 (18)	0.016 (2)	0.0125 (17)	−0.0021 (15)	−0.0025 (14)	−0.0031 (14)
C10	0.0284 (19)	0.019 (2)	0.0143 (17)	0.0016 (16)	−0.0038 (15)	0.0010 (15)
C11	0.0193 (17)	0.021 (2)	0.0172 (18)	0.0057 (15)	−0.0021 (14)	0.0007 (15)
C12	0.0168 (17)	0.019 (2)	0.0157 (17)	0.0019 (15)	0.0002 (14)	−0.0026 (14)
C13	0.0139 (17)	0.025 (2)	0.0135 (17)	0.0044 (15)	−0.0012 (14)	0.0009 (15)
C14	0.0147 (17)	0.029 (2)	0.0163 (18)	0.0011 (16)	−0.0010 (14)	−0.0068 (15)
C15	0.0174 (18)	0.022 (2)	0.0231 (19)	−0.0041 (15)	0.0027 (15)	−0.0117 (16)
C16	0.0153 (17)	0.019 (2)	0.0195 (18)	−0.0003 (15)	0.0032 (14)	−0.0038 (15)
C17	0.0127 (17)	0.019 (2)	0.0148 (17)	0.0025 (14)	0.0038 (14)	−0.0014 (14)
C18	0.0191 (19)	0.015 (2)	0.030 (2)	0.0010 (14)	0.0018 (15)	−0.0055 (16)
C19	0.028 (2)	0.019 (2)	0.023 (2)	−0.0039 (17)	−0.0014 (16)	−0.0033 (16)
C20	0.0235 (19)	0.025 (2)	0.0156 (18)	0.0052 (16)	−0.0017 (15)	−0.0019 (15)

### Geometric parameters (Å, º)

**Table:**

Fe1—N1	1.977 (3)	C2—C3	1.372 (5)
Fe1—N1^i^	1.977 (3)	C2—H2	0.9500
Fe1—N2	1.974 (3)	C3—C4	1.408 (5)
Fe1—N2^i^	1.974 (3)	C3—H3	0.9500
Fe1—N3	1.966 (3)	C4—C5	1.415 (4)
Fe1—N3^i^	1.966 (3)	C4—C6	1.439 (5)
S1—O1	1.417 (3)	C5—C8	1.414 (5)
S1—O2	1.429 (3)	C6—C7	1.341 (5)
S1—N4	1.581 (3)	C6—H6	0.9500
S1—C19	1.827 (4)	C7—C9	1.439 (5)
S2—O3	1.431 (2)	C7—H7	0.9500
S2—O4	1.435 (2)	C8—C9	1.403 (5)
S2—N4	1.574 (3)	C9—C10	1.413 (5)
S2—C20	1.824 (4)	C10—C11	1.368 (5)
N1—C1	1.341 (4)	C10—H10	0.9500
N1—C5	1.366 (4)	C11—C12	1.388 (5)
N2—C8	1.364 (4)	C11—H11	0.9500
N2—C12	1.326 (4)	C12—H12	0.9500
N3—C13	1.343 (4)	C13—C14	1.400 (5)
N3—C17	1.368 (4)	C13—H13	0.9500
O5—H5W	0.875 (10)	C14—C15	1.360 (5)
F1—C19	1.318 (4)	C14—H14	0.9500
F2—C19	1.324 (4)	C15—C16	1.410 (5)
F3—C19	1.329 (4)	C15—H15	0.9500
F4—C20	1.327 (4)	C16—C17	1.401 (5)
F5—C20	1.330 (4)	C16—C18	1.439 (5)
F6—C20	1.327 (4)	C17—C17^i^	1.425 (6)
C1—C2	1.400 (4)	C18—C18^i^	1.345 (7)
C1—H1	0.9500	C18—H18	0.9500
			
N3—Fe1—N3^i^	83.17 (16)	C7—C6—C4	121.7 (3)
N3—Fe1—N2^i^	174.17 (11)	C7—C6—H6	119.1
N3^i^—Fe1—N2^i^	94.38 (11)	C4—C6—H6	119.1
N3—Fe1—N2	94.38 (11)	C6—C7—C9	121.1 (3)
N3^i^—Fe1—N2	174.17 (11)	C6—C7—H7	119.5
N2^i^—Fe1—N2	88.54 (16)	C9—C7—H7	119.5
N3—Fe1—N1^i^	92.05 (11)	N2—C8—C9	123.7 (3)
N3^i^—Fe1—N1^i^	89.01 (11)	N2—C8—C5	116.3 (3)
N2^i^—Fe1—N1^i^	82.60 (11)	C9—C8—C5	120.0 (3)
N2—Fe1—N1^i^	96.38 (11)	C8—C9—C10	116.6 (3)
N3—Fe1—N1	89.01 (11)	C8—C9—C7	118.7 (3)
N3^i^—Fe1—N1	92.05 (11)	C10—C9—C7	124.7 (3)
N2^i^—Fe1—N1	96.38 (11)	C11—C10—C9	119.2 (3)
N2—Fe1—N1	82.60 (11)	C11—C10—H10	120.4
N1^i^—Fe1—N1	178.58 (17)	C9—C10—H10	120.4
O1—S1—O2	118.91 (16)	C10—C11—C12	120.1 (3)
O1—S1—N4	108.49 (16)	C10—C11—H11	120.0
O2—S1—N4	116.70 (16)	C12—C11—H11	120.0
O1—S1—C19	103.70 (17)	N2—C12—C11	122.9 (3)
O2—S1—C19	104.80 (16)	N2—C12—H12	118.6
N4—S1—C19	101.81 (17)	C11—C12—H12	118.6
O3—S2—O4	118.49 (16)	N3—C13—C14	122.6 (3)
O3—S2—N4	116.61 (16)	N3—C13—H13	118.7
O4—S2—N4	108.02 (16)	C14—C13—H13	118.7
O3—S2—C20	104.65 (16)	C15—C14—C13	119.8 (3)
O4—S2—C20	103.83 (15)	C15—C14—H14	120.1
N4—S2—C20	103.19 (17)	C13—C14—H14	120.1
C1—N1—C5	117.1 (3)	C14—C15—C16	120.1 (3)
C1—N1—Fe1	129.7 (2)	C14—C15—H15	120.0
C5—N1—Fe1	112.9 (2)	C16—C15—H15	120.0
C12—N2—C8	117.4 (3)	C17—C16—C15	116.6 (3)
C12—N2—Fe1	129.9 (2)	C17—C16—C18	118.2 (3)
C8—N2—Fe1	112.6 (2)	C15—C16—C18	125.2 (3)
C13—N3—C17	117.1 (3)	N3—C17—C16	123.9 (3)
C13—N3—Fe1	130.2 (2)	N3—C17—C17^i^	115.67 (18)
C17—N3—Fe1	112.7 (2)	C16—C17—C17^i^	120.4 (2)
S1—N4—S2	124.92 (19)	C18^i^—C18—C16	121.4 (2)
N1—C1—C2	123.0 (3)	C18^i^—C18—H18	119.3
N1—C1—H1	118.5	C16—C18—H18	119.3
C2—C1—H1	118.5	F1—C19—F2	107.8 (3)
C3—C2—C1	119.7 (3)	F1—C19—F3	108.7 (3)
C3—C2—H2	120.1	F2—C19—F3	107.6 (3)
C1—C2—H2	120.1	F1—C19—S1	111.6 (3)
C2—C3—C4	119.5 (3)	F2—C19—S1	111.1 (3)
C2—C3—H3	120.2	F3—C19—S1	109.9 (3)
C4—C3—H3	120.2	F4—C20—F5	108.1 (3)
C3—C4—C5	116.9 (3)	F4—C20—F6	108.5 (3)
C3—C4—C6	125.4 (3)	F5—C20—F6	108.6 (3)
C5—C4—C6	117.6 (3)	F4—C20—S2	111.0 (3)
N1—C5—C8	115.5 (3)	F5—C20—S2	108.8 (2)
N1—C5—C4	123.6 (3)	F6—C20—S2	111.8 (2)
C8—C5—C4	120.8 (3)		

Symmetry code: (i) −*x*+1, *y*, −*z*+1/2.

### Hydrogen-bond geometry (Å, º)

**Table:**

*D*—H···*A*	*D*—H	H···*A*	*D*···*A*	*D*—H···*A*
O5—H5*W*···O4^ii^	0.88 (1)	2.23 (6)	2.963 (4)	141 (7)
C2—H2···O3^iii^	0.95	2.50	3.433 (4)	166
C14—H14···O2	0.95	2.53	3.481 (5)	174

Symmetry codes: (ii) *x*−1/2, *y*−1/2, *z*; (iii) −*x*+1, −*y*+2, −*z*+1.
