# Supplementary figures and images for: Crystal structure of tris­(1,10-phenanthroline-κ2 N,N′)iron(II) bis­[bis­(tri­fluoro­methyl­sulfon­yl)imide] monohydrate
Source: Acta Crystallogr E Crystallogr Commun. 2015 Jan 1;71(Pt 1):m8–9. doi: 10.1107/S2056989014026966 (PMC4331886; doi:10.1107/S2056989014026966)

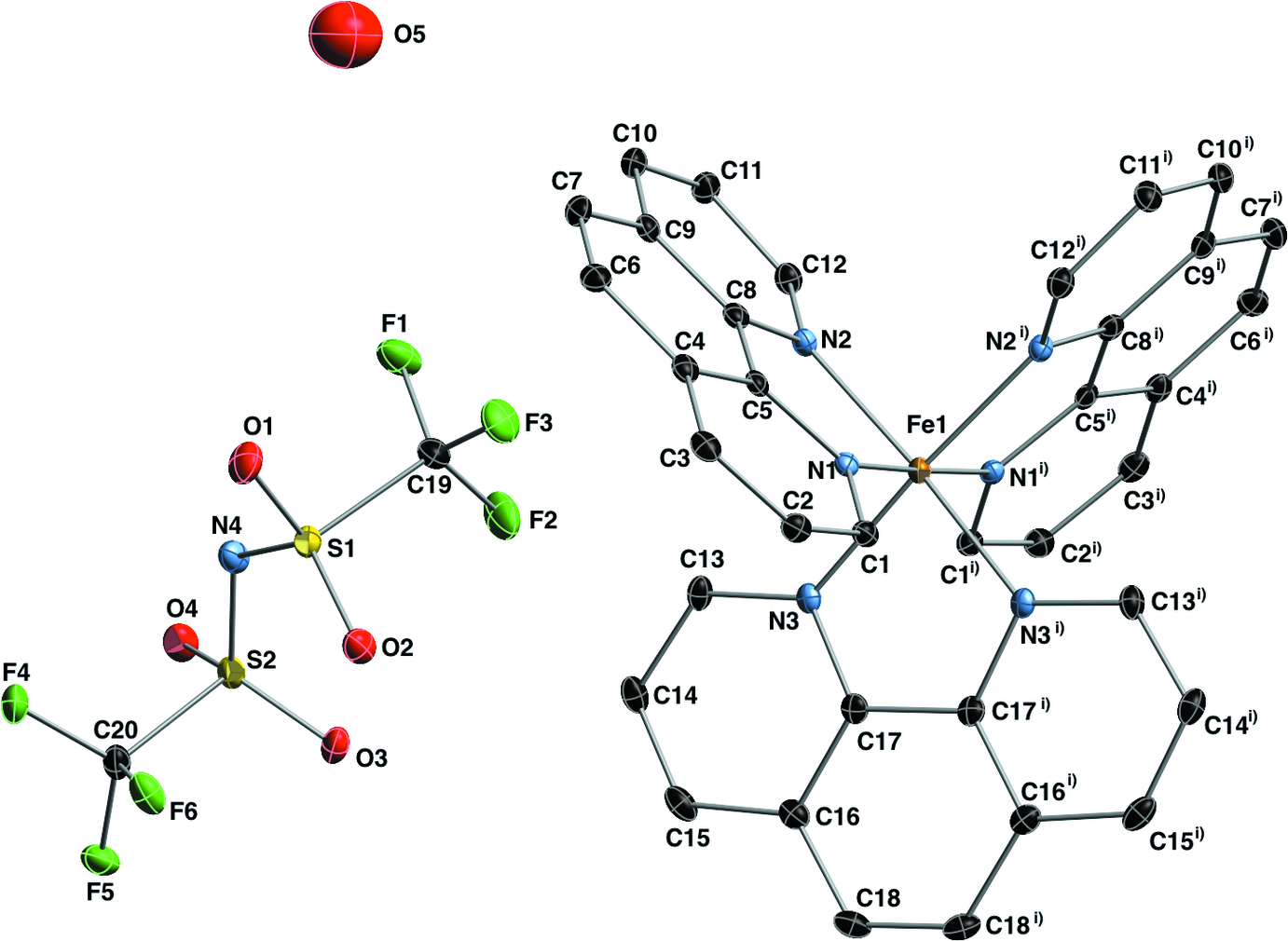

Supplement: Supplementary file 3 [file e-71-000m8-fig1.tif]

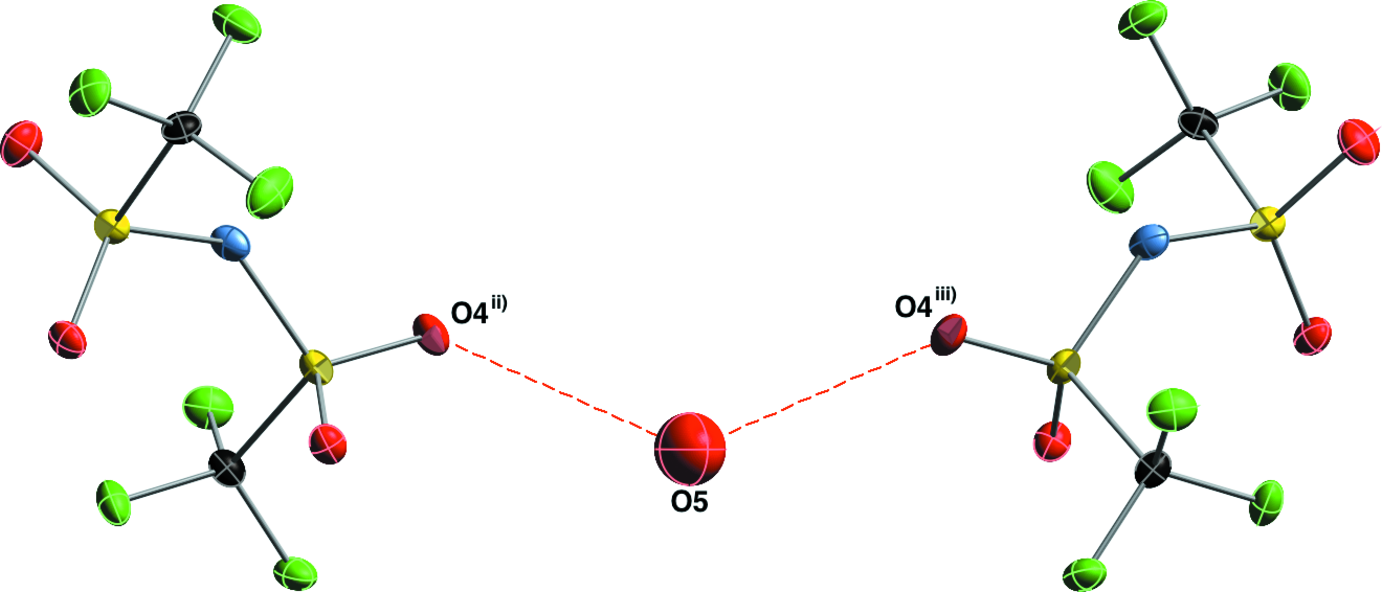

Supplement: Supplementary file 4 [file e-71-000m8-fig2.tif]
